# The use of first-generation cephalosporin antibiotics, cefalexin and cefradine, is not associated with induction of simulated *Clostridioides difficile* infection

**DOI:** 10.1093/jac/dkab349

**Published:** 2021-09-25

**Authors:** Anthony M Buckley, Ines B Moura, James Altringham, Duncan Ewin, Emma Clark, Karen Bentley, Vikki Wilkinson, William Spittal, Georgina Davis, Mark H Wilcox

**Affiliations:** 1 Healthcare-Associated Infections Group, Leeds Institute of Medical Research, Faculty of Medicine and Health, University of Leeds, Leeds, LS1 9JT, UK; 2 Microbiology, Leeds Teaching Hospitals NHS Trust, Old Medical School, Leeds General Infirmary, Leeds, LS1 3EX, UK

## Abstract

**Objectives:**

The use of broad-spectrum cephalosporins is associated with induction of *Clostridioides difficile* infection (CDI). Recent knowledge on the importance of the healthy microbiota in preventing pathogen colonization/outgrowth highlights the caution needed when prescribing broad-spectrum antibiotics. The use of historical narrow-spectrum antibiotics, such as first-generation cephalosporins, is gaining increased attention once more as they have a reduced impact on the microbiota whilst treating infections. Here, the effects of two first-generation cephalosporins, compared with a third-generation cephalosporin, on the human microbiota were investigated and their propensity to induce simulated CDI.

**Methods:**

Three *in vitro* chemostat models, which simulate the physiochemical conditions of the human colon, were seeded with a human faecal slurry and instilled with either narrow-spectrum cephalosporins, cefalexin and cefradine, or a broad-spectrum cephalosporin, ceftriaxone, at concentrations reflective of colonic levels.

**Results:**

Instillation of cefalexin was associated with reduced recoveries of *Bifidobacterium* and Enterobacteriaceae; however, *Clostridium* spp. recoveries remained unaffected. Cefradine exposure was associated with decreased recoveries of *Bifidobacterium* spp., *Bacteroides* spp. and Enterobacteriaceae. These changes were not associated with induction of CDI, as we observed a lack of *C. difficile* spore germination/proliferation, thus no toxin was detected. This is in contrast to a model exposed to ceftriaxone, where CDI was observed.

**Conclusions:**

These model data suggest that the minimal impact of first-generation cephalosporins, namely cefalexin and cefradine, on the intestinal microbiota results in a low propensity to induce CDI.

## Introduction

The intestinal microbiota exists in a symbiotic relationship with humans, where the microbiota aids digestion of foodstuffs, immune system regulation and protection from pathogenic organisms—a term called colonization resistance. There exists a balance in clinical practices between the need to treat patients with potentially unknown microbial infections and the need to reduce the impact of those antibiotics on the resident microbiota. Antibiotics are life-saving drugs; however, their use is associated with risks, such as consequences of healthcare-associated infections and antibiotic resistance. Whilst broad-spectrum antibiotics target a wide range of pathogenic bacteria, their use impacts upon the commensal gut microbiota.^1–3^ The reduction in microbial diversity, even if temporary, can increase the carriage of pathogenic microorganisms or those harbouring antibiotic-resistance determinants.^4^^,^^5^

*Clostridioides difficile* infection (CDI) is the leading cause of antibiotic-associated diarrhoea and causes significant morbidity and mortality worldwide. CDI is highly associated with the use of antibiotics, where the antimicrobial action depletes the intestinal microbiota, which allows germination and outgrowth of ingested *C. difficile* spores. The financial burden of CDI cases on healthcare systems is estimated to be €3 billion in Europe and $4.8 billion in the USA.^6–8^ Broad-spectrum antibiotics, such as amoxicillin, are the most highly prescribed antibiotics and are highly associated with induction of CDI.^3^^,^^9^ Indeed, the use of second-, third- and fourth-generation cephalosporins is associated with a significantly increased risk of developing CDI; however, there is a low risk reported with first-generation cephalosporin prescriptions.^10^ The use of narrow-spectrum antibiotics has gained attention in recent years, where they have reduced impact on the microbiota, already have approval for human medical use and are inexpensive.

Cefalexin and cefradine are first-generation cephalosporins used to treat respiratory or urinary tract infections, with activity mostly against Gram-positive cocci and some Gram-negative organisms, including *Streptococcus* spp., *Escherichia coli and Proteus* spp.^11^ Like all cephalosporins, cefalexin and cefradine bind and inactivate the bacterial penicillin-binding proteins (PBPs). Inhibition of PBPs prevents the cross-linking of the peptidoglycan layer, which results in weakened bacterial cell walls and, ultimately, cell lysis. However, these antibiotics are susceptible to different β-lactamases. We have previously shown, using an *in vitro* human gut model, that ceftriaxone affects the indigenous microbiota, specifically the recoveries of *Lactobacillus* spp., *Clostridium* spp. and *Enterococcus* spp., which provides a niche for the onset of simulated CDI.^3^ Here, using this *in vitro* human gut model, we assessed the impact of cefalexin and cefradine on a healthy microbiota and the propensity to induce CDI, alongside a comparator antibiotic, ceftriaxone.^3^ This model consists of three chemostat vessels arranged in a weir cascade fashion, where each vessel mimics the physiological conditions of the proximal to distal colon.^12^

## Materials and methods

### Gut model

#### Setup

Three triple-staged gut models were assembled to simulate CDI induction, as previously described.^12^^,^^13^ Briefly, each model was composed of three chemostat vessels and maintained at physiological conditions; vessel 1 (pH 5.5 ± 0.1, 280 mL; proximal colon), vessel 2 (pH 6.2 ± 0.1, 300 mL; medial colon) and vessel 3 (pH 6.7 ± 0.1, 300 mL; distal colon). An anaerobic environment was maintained by sparging each vessel with nitrogen, and a complex growth medium connected to vessel 1 at a preestablished rate of 0.015 L/h.^13^

Faecal samples from five healthy donors (aged ≥60 years old with no history of antimicrobial usage in the previous 6 months) were individually screened for the presence of glutamine dehydrogenase (GDH), a constitutively expressed *C. difficile*-specific protein, as determined by the EIA C. DIFF CHEK^TM^ - 60 test (TECHLAB Inc., USA). The ages of participants who provided faecal donations were chosen to represent a common risk factor for CDI.^14^ Each donor faecal sample was screened as negative for *C. difficile* by EIA C. DIFF CHEK^TM^. Following this, faecal samples were diluted 1:10 with pre-reduced PBS. This slurry was filtered to remove large particulate matter. Each vessel, of each model, was seeded with ∼160 mL of this slurry to start the experiment. A small aliquot of faecal slurry was kept anaerobically at 37°C and the bacterial populations were enumerated (as described below).

#### Ethics

The collection and use of human faeces in our gut model has been approved by the School of Medicine Research Ethics Committee, University of Leeds (MREC 15-070; Investigation of the Interplay between Commensal Intestinal Organisms and Pathogenic Bacteria). Participants were provided with a ‘Participant Information Sheet’ (PIS) detailing a lay summary of the *in vitro* gut model and the scientific work they are contributing to by providing a faecal donation. Within this PIS, it is explained that by providing the sample the participant is giving informed consent for that sample to be used in the gut model.

#### Experimental design

**Figure 1.**
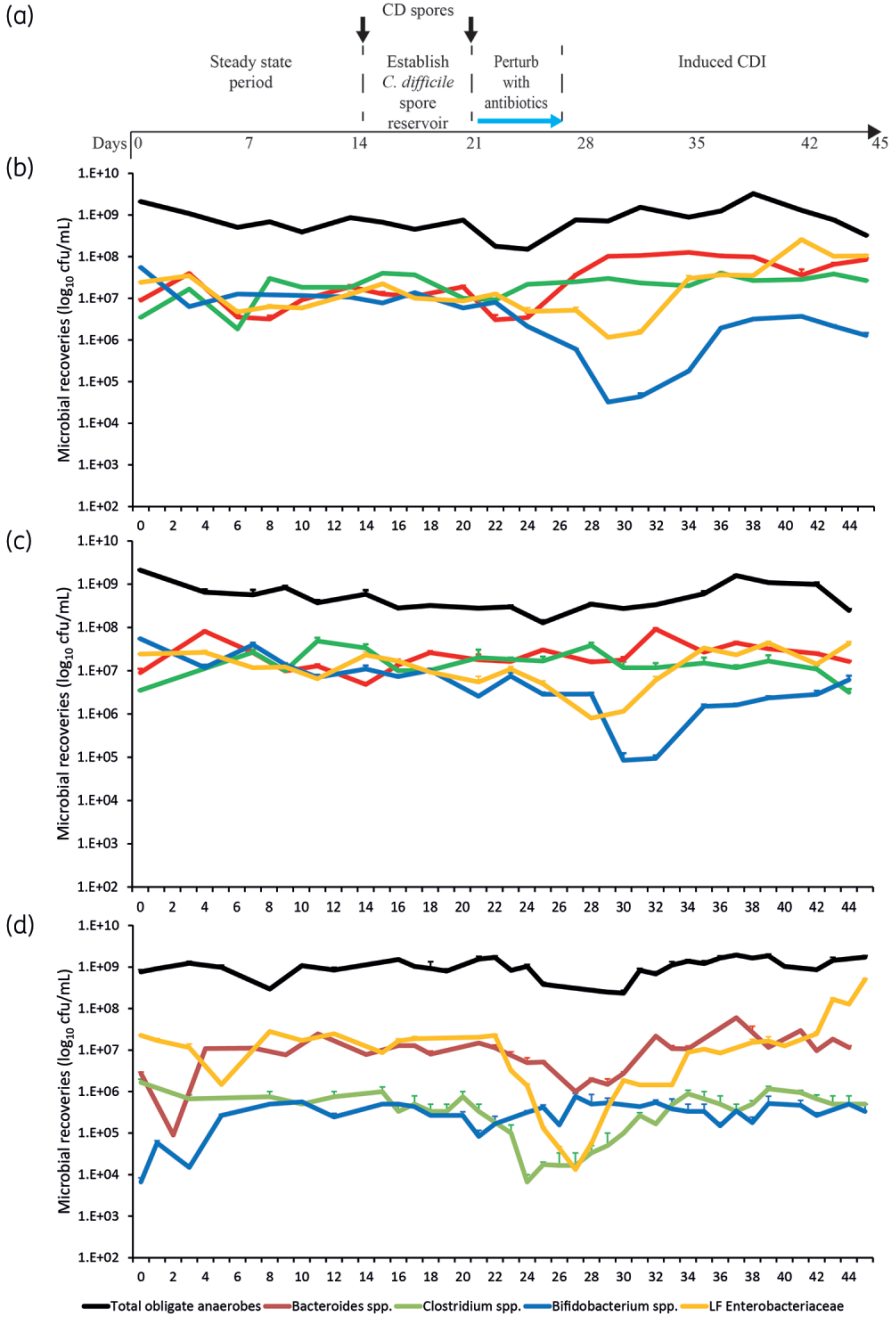
Schematic timeline of the *in vitro* triple-stage chemostat gut model and experimental design for each model (a). *C. difficile* (CD) spores were added to each model (black arrows) before addition of antibiotics (blue arrow). Microbial populations were monitored after exposure to either cefalexin (b), cefradine (c) or ceftriaxone (d). The total obligate anaerobic bacteria (black lines), *Bacteroides* spp. (red lines), *Bifidobacterium* spp. (blue lines), *Clostridium* spp. (green lines) and lactose-fermenting (LF) Enterobacteriaceae (orange lines) are shown as mean log_10_ cfu/mL from three technical replicates. Limit of detection for this assay is 1.2 log_10_ cfu/mL.

The experimental timeline for these models is depicted in Figure 1a. After addition of the faecal slurry, microbial populations were monitored for 14 days, without further intervention, to ensure the populations reached steady state. A 1 mL aliquot of *C. difficile* spores (10^7^ spores/mL) of strain 210 [BI/NAP1/PCR ribotype (RT) 027/toxinotype III]^15^ was added to vessel 1 of each model. This was done to establish that the microbiota had formed colonization resistance against *C. difficile* germination. One week later, another dose of *C. difficile* spores was added to the model and each model was instilled with either cefalexin (15 mg/L, four times daily for 7 days), cefradine (15 mg/L, four times daily for 7 days) or ceftriaxone (150 mg/L, once daily for 7 days) and the microbial populations monitored for 2 weeks after the last dose. These antibiotic concentrations are reflective of the concentration found in the human colon.^3^^,^^11^^,^^16^ Bacterial populations, including *C. difficile*, were monitored daily throughout the experiment using selective and non-selective agars, as outlined previously.^12^ Only those counts from vessel 3 are shown.

### Preparation of C. difficile RT027 strain 210 spores

*C. difficile* spores for gut model inoculation were prepared as previously described.^17^ Briefly, *C. difficile* RT027 was grown in brain heart infusion (BHI) broth anaerobically at 37°C for 6 days and removed from the incubator and incubated aerobically at room temperature overnight to further induce sporulation. Growth was harvested by centrifugation and incubated with PBS supplemented with 10 mg/mL lysozyme at 37°C overnight. Samples were separated using a sucrose gradient and spores were treated with PBS supplemented with 20 ng/mL protease K and 200 nM EDTA. Spores were then separated using a sucrose gradient and washed with PBS twice before a final resuspension in 30 mL. These were enumerated and diluted to approximately 1 × 10^7^ spores/mL for use in the models.^18^

### Enumeration of endogenous bacteria and quantification of C. difficile toxin

Gut microbiota populations were monitored using viable enumeration on selective and non-selective agars, as described previously.^12^ Microbial colonies were enumerated and identified, based on colony morphology and MALDI-TOF identification. Each bacterial population was measured in triplicate (three technical replicates of a single biological replicate) in vessels 2 and 3. *C. difficile* total viable counts (TVCs) and spore counts were measured from all vessels; spore counts were obtained through plating serial dilutions of model fluid after alcohol shock. The limits of detection for TVCs or spore counts were 1.2 or l.5 log_10_ cfu/mL, respectively.

*C. difficile* cytotoxin was monitored using a semi-quantitative Vero cell cytotoxicity assay as previously described.^12^ Cytotoxin titre was expressed as log_10_ relative units (RU) at the highest dilution with >70% cell rounding, i.e. 10°, 1 RU; 10^1^, 2 RU etc.

### Antibiotic bioassays

The concentration of ceftriaxone in each vessel was determined by antibiotic bioassay, as previously described; *E. coli* ATCC 25922 was inoculated onto Mueller–Hinton agar (Oxoid, UK).^3^ Cefalexin and cefradine concentrations were determined by antibiotic bioassay, as before except the *E. coli* ATCC 25922 indicator strain was incorporated into Iso-Sensitest agar (Oxoid, UK) and either cefalexin or cefradine calibrator curve concentrations (range between 128 and 0.5 mg/L) were added randomly to each plate. The limits of detection were 0.05 mg/L for cefalexin and cefradine, and 1 mg/L for ceftriaxone.

## Results

### Formation of colonization resistance against C. difficile spores

Upon inoculation, the microbial populations were allowed to equilibrate for 2 weeks prior to further intervention; during this time, microbial growth patterns settled. To determine whether the established microbiota within each model conferred colonization resistance against *C. difficile* spore germination, we exposed each model to 10^7^ spores/mL and monitored for germination and outgrowth. Once added to the model, *C. difficile* cells remained in spore form, where no evidence of germination was seen. Formation of colonization resistance against *C. difficile* spore germination in the models ensures that any post-antibiotic induction of CDI would be directly related to the effects of the different antibiotics rather than microbiota stability within the model.

### Antibiotic-induced changes to gut microbiota populations

#### Cefalexin

Cefalexin instillation caused declines to the *Bifidobacterium* spp. (2.4 log_10_ cfu/mL) and lactose-fermenting Enterobacteriaceae (1.0 log_10_ cfu/mL) recoveries, where *Bifidobacterium* spp. failed to recover to pre-antibiotic levels, whilst lactose-fermenting Enterobacteriaceae recovered to levels approximately 1 log_10_ cfu/mL higher than pre-antibiotic levels (Figure 1b). Cefalexin promoted an initial decline in *Bacteroides* spp.; however, this population soon recovered to approximately 1 log_10_ cfu/mL higher than pre-antibiotic levels. *Lactobacillus* spp. also declined by 1.2 log_10_ cfu/mL after exposure to cefalexin but recovered after withdrawal of the antibiotic. There was no change in *Enterococcus* spp., and *Clostridium* spp. remained unchanged throughout the experiment.

#### Cefradine

Cefradine instillation caused similar declines to the *Bifidobacterium* spp. (2.0 log_10_ cfu/mL) and lactose-fermenting Enterobacteriaceae (1.1 log_10_ cfu/mL) populations compared with pre-antibiotic levels (Figure 1c). These bacterial populations recovered to pre-antibiotic levels by Day 46. Interestingly, *Bacteroides* spp. and *Clostridium* spp. remained unaffected by the antibiotic levels. *Enterococcus* spp. recoveries increased by 1.0 log_10_ cfu/mL after exposure to cefradine but decreased to pre-antibiotic levels by Day 46.

#### Ceftriaxone

Instillation of ceftriaxone caused widespread changes to the microbiota. Lactose-fermenting Enterobacteriaceae decreased by 3.2 log_10_ cfu/mL during ceftriaxone instillation; however, post-antibiotic Enterobacteriaceae increased by 1.4 log_10_ cfu/mL compared with pre-antibiotic levels (Figure 1d). *Bacteroides* spp. and *Clostridium* spp. showed decreases of 1.2 and 1.4 log_10_ cfu/mL, respectively, during ceftriaxone exposure; however, these populations recovered to pre-antibiotic levels 2 weeks after withdrawal. *Enterococcus* spp. were unaffected by ceftriaxone instillation; however, there was a 3.9 log_10_ cfu/mL increase during the dysbiosis created after antibiotic exposure. Interestingly, *Bifidobacterium* spp. remained unaffected by ceftriaxone (Figure 1d).

**Figure 2.**
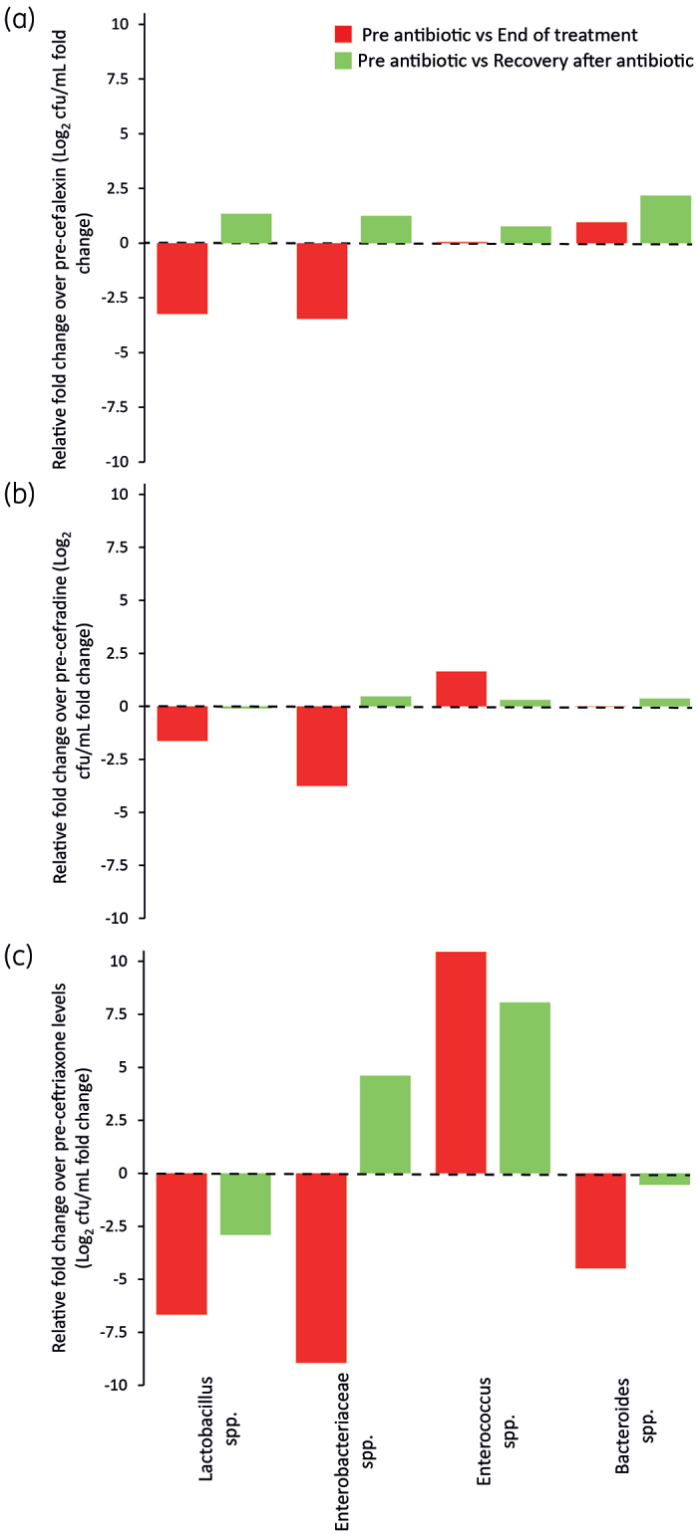
Comparative effects of cefalexin (a), cefradine (b) and ceftriaxone (c) on the human gut microbiota. Key bacterial members of the microbiota were enumerated just before antibiotics were added, when the antibiotic treatment ended and 2 weeks after antibiotic withdrawal. Results shown are the fold change in bacterial recovery of the end of antibiotic treatment (red bars) and 2 weeks after antibiotic (green bars) compared with pre-antibiotic levels.

Comparing the effects of each antibiotic on the microbiota showed that cefalexin and cefradine caused the least disruption to the microbiota compared with ceftriaxone. The disruption caused by cefalexin and cefradine was characterized by reductions of −3.5-fold and −3.8-fold, respectively, to the Enterobacteriaceae population compared with pre-antibiotic levels (Figure 2a and b). *Lactobacillus* was also affected during cefalexin and cefradine instillation, showing reductions of −3.2-fold and −1.6-fold, respectively. However, all microbial populations had recovered to pre-antibiotic levels by the end of the experiment. Ceftriaxone caused the most disruption to the microbiota, with *Lactobacillus*, Enterobacteriaceae and *Bacteroides* populations showing −6.8-fold, −8.9-fold and −4.5-fold decreases compared with pre-antibiotic levels. The microbial dysbiosis observed was maintained for at least 2 weeks after withdrawal, with Enterobacteriaceae and *Enterococcus* spp. 4.6-fold and 8.1-fold higher than pre-ceftriaxone levels (Figure 2c).

### Antibiotic disruption of the gut microbiota does not induce CDI

**Figure 3.**
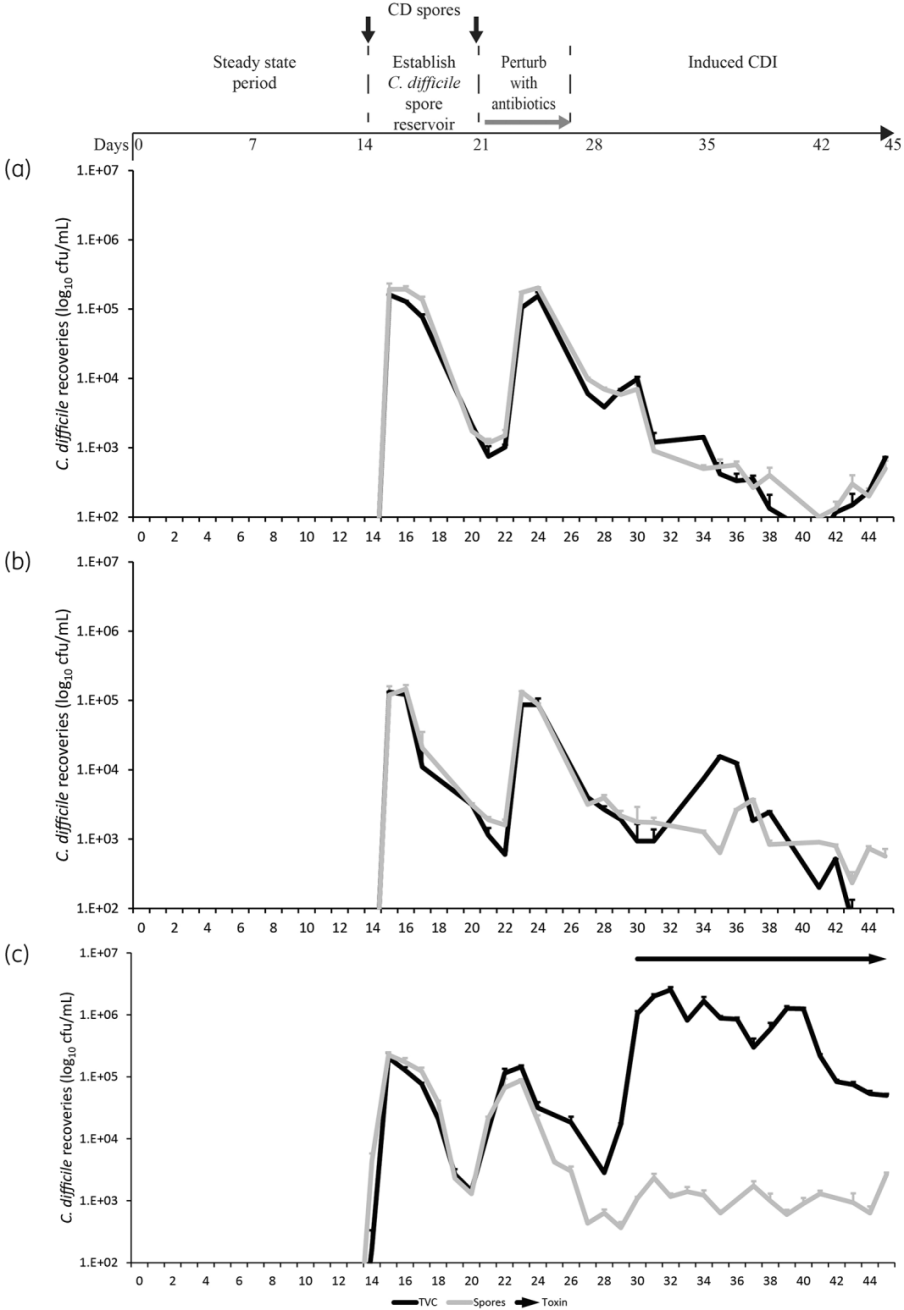
Recovery of *C. difficile* populations in vessel 3 after instillation of either cefalexin (a), cefradine (b) or ceftriaxone (c). The *C. difficile* TVCs (black lines) and spore populations (grey lines) are shown as mean log_10_ cfu/mL from three technical replicates. Toxin production (arrows) was measured by cell toxicity assay—toxin activity was only detected after exposure to ceftriaxone (c); thus, no arrow is visible in (a) and (b). Limits of detection for this assay are 1.2 and 2.5 log_10_ cfu/mL for total viable and spores counts, respectively.

Instillation of cefalexin, cefradine or ceftriaxone reached peak concentrations of 4.8, 5.3 and 39.0 mg/L, respectively, in vessel 1 of each model; antibiotic concentrations were lower in vessels 2 and 3 in each model. These concentrations of cefalexin and cefradine achieved in our gut model did not cause simulated CDI. There was no divergence between *C. difficile* TVCs and spore recoveries after cefalexin exposure and, whilst there was limited germination after cefradine exposure, there was no toxin detected in these models (Figure 3a and b). The level of ceftriaxone-mediated microbiota disruption induced simulated CDI where the TVCs diverged from the spore counts, with peak *C. difficile* recoveries occurring 5 days after antibiotic instillation (Day 33; 6.4 log_10_ cfu/mL), which was preceded by toxin detection. Peak toxin was detected on Day 35 at 4.5 RU toxin activity (Figure 3c).

## Discussion

The toxicity and tolerability profiles of cephalosporins make them ideal treatment options for a wide variety of infections; however, cephalosporin-resistant pathogens can exploit this and cause serious complications. *C. difficile* is one such pathogen, where the use of second- to fourth-generation cephalosporins is associated with a high OR of inducing CDI (OR between 3.2 and 2.14), whilst the use of first-generation cephalosporins has a lower OR of 1.36.^10^ The lower risk of developing subsequent CDI after first-generation cephalosporin use is likely due to the narrow spectrum of activity against bacterial populations.^11^ In our study, we showed that cefalexin and cefradine temporarily reduced the Enterobacteriaceae and *Lactobacillus* populations, which recovered to pre-antibiotic levels soon after withdrawal. In contrast, ceftriaxone caused more disruption to the microbiota, where, 2 weeks after antibiotic withdrawal, overgrowth of opportunistic pathogens Enterobacteriaceae and *Enterococcus* spp. was seen, and lower levels of *Lactobacillus* spp., compared with pre-antibiotic levels. The observed effects of ceftriaxone on the microbiota were similar to those previously reported by our group and in clinical trials.^3^^,^^19^ This level of ceftriaxone-induced dysbiosis created a niche that allowed *C. difficile* spore germination and the onset of simulated CDI, whereas cefalexin and cefradine did not induce CDI.

Environmental metabolites, such as primary bile acids and some amino acids, act as cues for disease initiation during CDI, whereas other metabolites, such as ethanolamine and succinate, are utilized during vegetative growth.^20–22^ Antibiotic depletion of some *Clostridium* spp., such as *Clostridium scindens*, can result in accumulation of primary bile acids, which are a known germination signal for *C. difficile* spores, thus initiating disease.^23^ However, *Clostridium* spp. recoveries before, during and after first-generation cephalosporin exposure remained unchanged, suggesting that the capacity to metabolize primary bile acids may have been preserved after exposure to either first-generation cephalosporin tested. In contrast, *Clostridium* spp. were reduced by ceftriaxone, which could have caused increased concentrations of spore germinants in our system. A reduction in *Clostridium* spp. and *Bifidobacterium* spp. was seen with other cephalosporins (ceftaroline/cefotaxime), which induced simulated CDI in our model system.^3^^,^^13^ Upon germination, *C. difficile* still must compete with the microbiota for nutrients to grow. Interestingly, we did observe limited germination and outgrowth after cefradine instillation; however, there appeared to be sufficient microbial competition that prevented sufficient vegetative growth, with no toxin being detected. It has been shown that some *Clostridium* spp., such as *Clostridium bifermentans*, can actively compete with *C. difficile* by utilizing the bioavailable amino acid pool, thus preventing CDI.^24^ Thus, the differential effect of cefalexin/cefradine versus ceftriaxone on the *Clostridium* spp. could impact *C. difficile* growth and the different propensities to induce simulated CDI. The number of different classes of antimicrobials that are high risk for inducing CDI suggests that *C. difficile* can metabolically adapt to different niches created during antibiotic-mediated disruption of the microbiota.^25^

Whilst treating the primary infection, some classes of antibiotics can subsequently lead to life-threatening complications, such as CDI. There exists a need to determine the effect of different antibiotics upon the intestinal microbiota, as preservation of a patient’s microbiota should be incorporated in the multifaceted decision-making process when choosing a treatment option. Here, we show that two narrow-spectrum cephalosporins, cefalexin and cefradine, have a limited effect on the human microbiota and this disruption represents a low risk for subsequently developing simulated CDI. Our *in vitro* data support the clinical findings previously reported by Slimings and Riley.^10^

## References

[1] Nord CE , Brismar B, Kasholm-Tengve B et al Effect of piperacillin/tazobactam therapy on intestinal microflora. Scand J Infect Dis 1992; 24: 209–13. 1322559 10.3109/00365549209052614

[2] Zimmermann P , Curtis N. The effect of antibiotics on the composition of the intestinal microbiota - a systematic review. J Infect 2019; 79: 471–89. 31629863 10.1016/j.jinf.2019.10.008

[3] Baines SD , Chilton CH, Crowther GS et al Evaluation of antimicrobial activity of ceftaroline against *Clostridium difficile* and propensity to induce *C. difficile* infection in an *in vitro* human gut model. J Antimicrob Chemother 2013; 68: 1842–9. 23557928 10.1093/jac/dkt107

[4] Rooney CM , Sheppard AE, Clark E et al Dissemination of multiple carbapenem resistance genes in an *in vitro* gut model simulating the human colon. J Antimicrob Chemother 2019; 74: 1876–83. 30989197 10.1093/jac/dkz106

[5] Buckley AM , Altringham J, Clark E et al Eravacycline, a novel tetracycline derivative, does not induce *Clostridioides difficile* infection in an *in vitro* human gut model. J Antimicrob Chemother 2021; 76: 171–8. 32929459 10.1093/jac/dkaa386

[6] Aguado JM , Anttila VJ, Galperine T et al Highlighting clinical needs in *Clostridium difficile* infection: the views of European healthcare professionals at the front line. J Hosp Infect 2015; 90: 117–25. 25842241 10.1016/j.jhin.2015.03.001

[7] Lessa FC , Mu Y, Bamberg WM et al Burden of *Clostridium difficile* infection in the United States. N Engl J Med 2015; 372: 825–34. 25714160 10.1056/NEJMoa1408913 PMC10966662

[8] Sheitoyan-Pesant C , Abou Chakra CN, Pépin J et al Clinical and healthcare burden of multiple recurrences of *Clostridium difficile* infection. Clin Infect Dis 2016; 62: 574–80. 26582748 10.1093/cid/civ958

[9] Wilcox MH , Chalmers JD, Nord CE et al Role of cephalosporins in the era of *Clostridium difficile* infection. J Antimicrob Chemother 2017; 72: 1–18. 27659735 10.1093/jac/dkw385 PMC5161048

[10] Slimings C , Riley TV. Antibiotics and hospital-acquired *Clostridium difficile* infection: update of systematic review and meta-analysis. J Antimicrob Chemother 2014; 69: 881–91. 24324224 10.1093/jac/dkt477

[11] Hartley CL , Clements HM, Linton KB. Effects of cephalexin, erythromycin and clindamycin on the aerobic Gram-negative faecal flora in man. J Med Microbiol 1978; 11: 125–35. 660638 10.1099/00222615-11-2-125

[12] Moura IB , Buckley AM, Ewin D et al Omadacycline gut microbiome exposure does not induce *Clostridium difficile* proliferation or toxin production in a model that simulates the proximal, medial, and distal human colon. Antimicrob Agents Chemother 2019; 63: e01581-18. 30455242 10.1128/AAC.01581-18 PMC6355569

[13] Freeman J , Neill FJO, Wilcox MH. Effects of cefotaxime and desacetylcefotaxime upon *Clostridium difficile* proliferation and toxin production in a triple-stage chemostat model of the human gut. J Antimicrob Chemother 2003; 52: 96–102. 12775682 10.1093/jac/dkg267

[14] Bartlett JG , Gerding DN. Clinical recognition and diagnosis of *Clostridium difficile* infection. Clin Infect Dis 2008; 46: S12–18. 18177217 10.1086/521863

[15] Chilton CH , Crowther GS, Baines SD et al *In vitro* activity of cadazolid against clinically relevant *Clostridium difficile* isolates and in an *in vitro* gut model of *C. difficile* infection. J Antimicrob Chemother 2014; 69: 697–705. 24128668 10.1093/jac/dkt411

[16] Bullen BR , Ramsden CH, Kester RC. Peroperative cephradine concentrations in the gall bladder wall and bile. Curr Med Res Opin 1982; 8: 5–8. 7105822 10.1185/03007998209109749

[17] Lawley TD , Croucher NJ, Yu L et al Proteomic and genomic characterization of highly infectious *Clostridium difficile* 630 spores. J Bacteriol 2009; 191: 5377–86. 19542279 10.1128/JB.00597-09 PMC2725610

[18] Buckley AM , Spencer J, Candlish D et al Infection of hamsters with the UK *Clostridium difficile* ribotype 027 outbreak strain R20291. J Med Microbiol 2011; 60: 1174–80. 21330415 10.1099/jmm.0.028514-0 PMC3167879

[19] Pletz MWR , Rau M, Bulitta J et al Ertapenem pharmacokinetics and impact on intestinal microflora, in comparison to those of ceftriaxone, after multiple dosing in male and female volunteers. Antimicrob Agents Chemother 2004; 48: 3765–72. 15388432 10.1128/AAC.48.10.3765-3772.2004 PMC521887

[20] Francis MB , Allen CA, Shrestha R et al Bile acid recognition by the *Clostridium difficile* germinant receptor, CspC, is important for establishing infection. PLoS Pathog 2013; 9: e1003356. 23675301 10.1371/journal.ppat.1003356 PMC3649964

[21] Ferreyra JA , Wu KJ, Hryckowian AJ et al Gut microbiota-produced succinate promotes *C. difficile* infection after antibiotic treatment or motility disturbance. Cell Host Microbe 2014; 16: 770–7. 25498344 10.1016/j.chom.2014.11.003 PMC4859344

[22] Nawrocki KL , Wetzel D, Jones JB et al Ethanolamine is a valuable nutrient source that impacts *Clostridium difficile* pathogenesis. Environ Microbiol 2018; 20: 1419–35. 29349925 10.1111/1462-2920.14048 PMC5903940

[23] Buffie CG , Bucci V, Stein RR et al Precision microbiome reconstitution restores bile acid mediated resistance to *Clostridium difficile*. Nature 2015; 517: 205–8. 25337874 10.1038/nature13828 PMC4354891

[24] Girinathan B , DiBenedetto N, Worley J et al The mechanisms of *in vivo* commensal control of *Clostridioides difficile* virulence. bioRxiv 2020; https://doi.org/10.1101/2020.01.04.894915.

[25] Hofmann JD , Otto A, Berges M et al Metabolic reprogramming of *Clostridioides difficile* during the stationary phase with the induction of toxin production. Front Microbiol 2018; 9: 1970. 30186274 10.3389/fmicb.2018.01970 PMC6110889

